# Pulmonary Embolism after Arthroscopic Bankart and Rotator Cuff Repair

**Published:** 2017-05-15

**Authors:** Joshua M. Matthews, Susan S. Wessel, Ryan C. Pate, Alexander CM. Chong

**Affiliations:** 1 Department of Orthopaedics, University of Kansas School of Medicine-Wichita, KS; 2 Department of Orthopaedics, Robert J. Dole VA Medical Center, Wichita, KS; 3 Department of Orthopaedics, Via Christi Health, Department of Graduate Medical Education, Wichita, KS

**Keywords:** pulmonary embolism, rotator cuff, arthroscopy, orthopedic surgery

## Introduction

Pulmonary embolism (PE) is a blockage in one of the pulmonary arteries in the lungs. Since PE almost always occurs in conjunction with deep vein thrombosis (DVT), these two conditions together refer as venous thromboembolism (VTE). PE after shoulder arthroscopy is unusual and rare (reported incidence rate: 0.01 – 0.06%)^1^–^3^, but potentially life threatening.^4^ All patients with PE have a case fatality rate of 7 – 11%.^5^ In the body of literature, there were less than 50 reported cases of DVT and PE after shoulder arthroscopy.^1^–^3^, ^6^–^26^ The majority involved patients with a significant risk factor or underlying predisposition toward VTE.^23^ This case report presents a patient with inflammatory bowel disease who developed a symptomatic PE involving the medial segment of the right middle lobe of lung after arthroscopic Bankart and rotator cuff repair following a traumatic shoulder dislocation.

## Case Report

A 55-year-old right-hand dominant male (body mass index: 30.5 kg/m^2^) presented with right shoulder pain and weakness with overhead activities. He had an anterior dislocation during a fall one week prior. At the time of injury, he only had shoulder dislocation without any other injuries; therefore, he underwent a reduction and was placed in a sling at a local emergency room with limited ambulation. Radiographic images showed a fracture in the glenoid; a computed tomography (CT) of the shoulder also was obtained. The patient had a history of ulcerative colitis and Grave’s disease.

**Figure 1:**
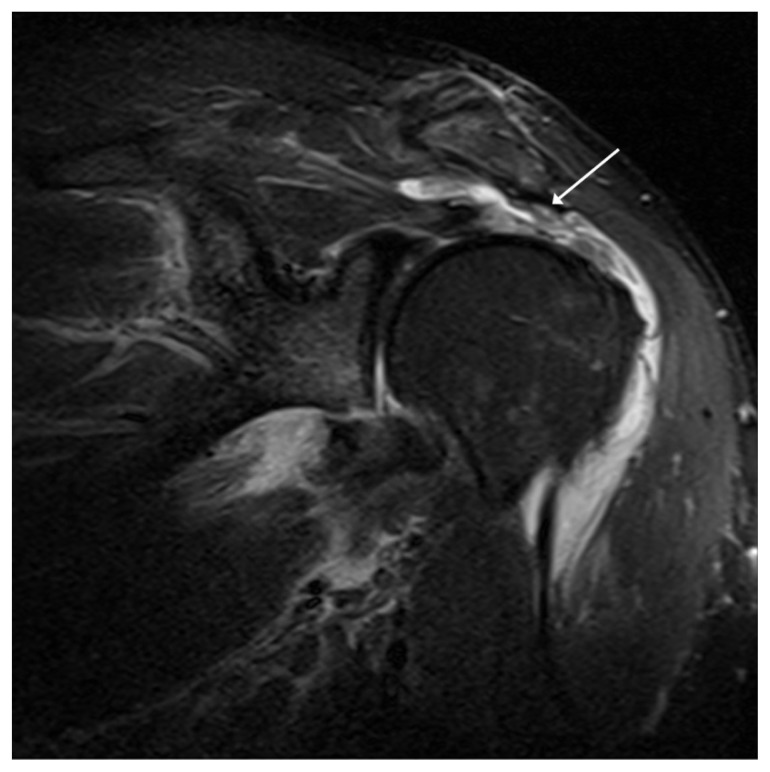
Magnetic resonance imaging (MRI) showed large retracted supraspinatus tendon avulsion following traumatic dislocation.

**Figure 2:**
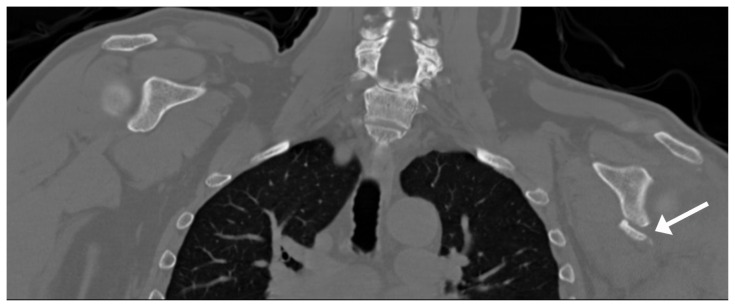
Computer tomography scan showed small anterior/inferior glenoid rim fracture (bony Bankart Lesion).

On physical examination, the patient’s right shoulder revealed pseudoparalysis with 3/5 supraspinatus strength. He was neurologically intact and stable with a normal apprehension test. Magnetic resonance imaging (MRI) and CT scan results showed a large full-thickness tear of the supraspinatus (Figure 1) and a small bony Bankart lesion on the anterior/inferior edge of the glenoid (Figure 2). It was recommended to repair the Bankart lesion and the rotator cuff arthroscopically.

An interscalene nerve block was performed prior to the surgery. No preoperative DVT chemoprophylaxis was administered. During the procedure, the patient was positioned in the lateral decubitus position and held in place with a beanbag. The operated limb was placed in 20° flexion and 45° abduction. Longitudinal traction was achieved with 4.5 kg (10 lbs.) weight to afford the best possible visualization of the joint.

**Figure 3:**
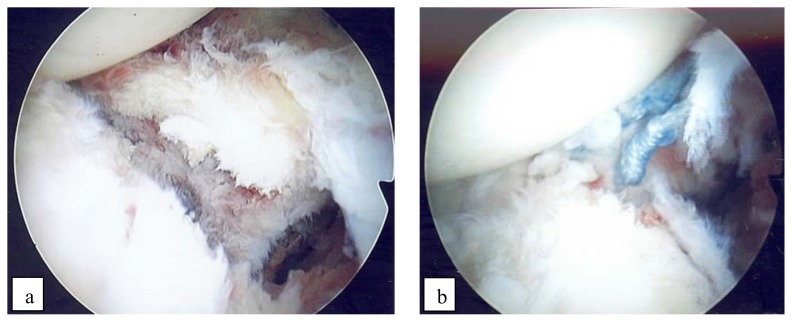
Intra-articular view of small bony Bankart lesion during surgery: (a) injury of the anterior glenoid labrum; (b) repaired Bankart lesion.

During surgery, the bony Bankart lesion was spotted (Figure 3a) and repair was performed. The operating surgeon attempted to incorporate the bony fragment by using suture anchors where the anchors fixed to the glenoid. There were difficulties, however, in navigating the suture passer around the piece of bone fragment. Due to the difficulty with the Bony Bankart repair, the surgery was longer than expected (total operative time: 3 hours and 15 minutes). Additionally, Sequential Compression Devices are not used routinely during shoulder arthroscopy at our institution.

As a result, a soft tissue Bankart repair was performed (Figure 3b). Following the Bankart lesion repair, the rotator cuff was addressed. A large tear of the supraspinatus was found and repaired using a suture-bridge double-row technique.

**Figure 4:**
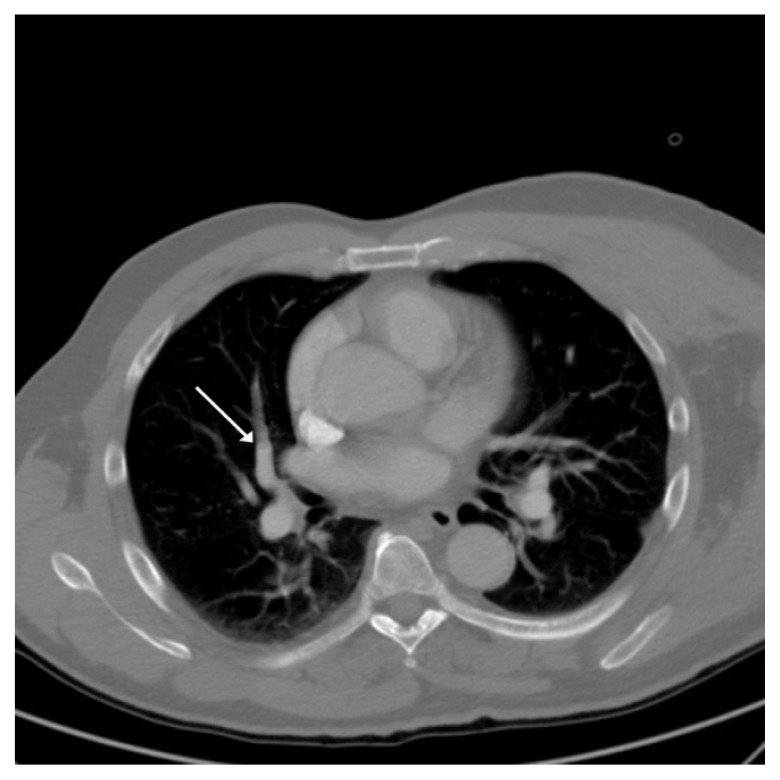
CT angiogram showed a PE involving the medial segment of the right middle lobe of lung.

After surgery, the affected right shoulder was immobilized in adduction and internal rotation, and the patient returned to most activities that did not include the use of his right shoulder. On post-operative day 4, the patient reported with shortness of breath, tachypnea, and tachycardia which began the night before. The patient, however, claimed no extremity pain or swelling and denied chest pain. A cardiac workup was performed and the results were normal; however, a D-dimer test was elevated. A CT angiogram showed a PE involving the medial segment of the right middle lobe of lung (Figure 4). A Doppler ultrasound test of all four extremities also was performed and the results were negative for DVT.

The patient was placed on the anticoagulation medications, enoxaparin and warfarin. His symptoms improved rapidly and he was discharged home four days later in good condition on warfarin therapy. At the two-week follow-up, he reported excellent satisfaction with the fixation. His pain was minimal and he was able to perform overhead activities. No further workup for thrombophilia was undertaken.

## Discussion

PE is an exceptionally rare yet serious complication after arthroscopic shoulder surgery.^1^,^12^ The exact incidence is unknown and only found in either case reports or small case series.^1^–^3^,^7^ ^8^ ^10^,^11^–^21^,^23^,^24^ Burkhart et al.^26^ was the first to report a case of a 32-year-old man who developed DVT in 1990 following shoulder arthroscopy, and Kim et al.^15^ was the first to report a case of a 45-year-old woman who developed a fatal PE in the contralateral axillary vein thrombosis after elective arthroscopic rotator cuff repair in 2010. Goldhober et al.^8^ presented a unique case of a 43-year-old female who developed a PE 41 days after repair of the rotator cuff, a distal clavicle excision, and a miniopen subpectoralis long head biceps tenodesis. Yamamoto et al.^3^ reported a 72-year-old female patient who developed a PE six days after arthroscopic rotator cuff repair.

Durant et al.^7^ performed a retrospective review of a single, fellowship trained orthopaedic surgeon for ten consecutive years and identified five cases of PE after arthroscopic rotator cuff repair, two of which were fatal. In that study, there were three females and two males with mean age of 61.4 years (range: 54 – 67 years) and the average time to diagnosis of a PE event following surgery was 6.8 days postoperatively (range: 3 – 18 days). Edgar et al.^11^ presented three cases of nonfatal PE following elective shoulder arthroscopy: 1) a 26-year-old male underwent arthroscopic debridement and revision labral repair; 2) a 45-year-old female underwent biceps tenotomy, rotator cuff repair, and subacromial decompression; and 3) a 59-year-old male underwent arthroscopic rotator cuff repair, biceps tenodesis, and subacromial decompression.

Schick et al.^10^ performed a retrospective case control review with 15,033 shoulder arthroscopy cases from 17 surgeons. The two study groups were the VTE group and the control group. Detailed information on each case of VTE was obtained through a review of surgical logs, including patient demographics, intraoperative details, any VTE prophylactic measures utilized or after surgery, and an extensive list of comorbidities and patient risk factors. Twenty-two patients of the 15,033 cases developed VTE (DVT: 15, PE: 8). Randelli et al.^16^ reported six patients (DVT: 5, PE: 1) who developed VTE from 9,385 arthroscopy surgeries by 59 orthopaedic surgeons. Kuremsky et al.^13^ reviewed 1,908 patients over five consecutive years that had undergone shoulder arthroscopy and reported five DVTs, four PEs, but no fatalities, with an overall thromboembolic complication rate of 0.31%. Hoxie et al.^19^ reviewed 1,176 patients who underwent operative procedures for rotator cuff tears, and three patients (0.26%) developed PE, 1, 7, and 30 days postoperatively. For this case report, this patient had been the operating surgeon’s only thromboembolic complication, which developed four days after arthroscopic Bankart lesion and rotator cuff repair, with a rate of 0.45%.

Arthroscopic shoulder surgery is safe with recent analysis reporting 30-day complication and readmission rates performed at 0.98%.^27^ The most common reason for readmission was PE (0.09%). Orthopedic surgeons should be aware of the possibility for delayed complications, be able to recognize sentinel symptoms, and should take the appropriate steps in delivering therapeutic care. If precautions are not taken and symptomatic patients are not screened appropriately, the consequences could be dreadful.

Given the extreme rarity of the condition and wide variety of presentations, PE cannot be diagnosed reliably based on history and clinical examination alone.^28^ Patients may present with upper extremity swelling, constitutional symptoms such as dyspnea, malaise, tachypnea, and tachycardia, pleuritic chest pain, discomfort with breathing, shortness of breath, and hypoxia as symptoms and signs of a potential VTE. However, most symptoms and signs of PE are not clinically obvious.^19^,^29^ Doppler ultrasound, spiral pulmonary CT angiogram, D-dimer tests, ventilation-perfusion lung scan, pulmonary artery angiography, and echocardiography (both transthoracic and transesophageal) are investigations of choice in patients with suspected PE.^3^,^7^,^8^,^15^,^19^,^30^,^31^ A CT angiogram is considered a criterion standard for a diagnosis of PE, but no single test is both sensitive and specific.^30^,^32^

In a studies performed by Turkstra et al.^33^ and Cortés et al.^21^, ultrasonography in patients with angiographically-proven PE had only 29% sensitivity in detecting venous thrombi in the extremities. Yamamoto et al.^3^ recommended serial D-dimer measurements in the perioperative period for detecting DVT/PE even in the arthroscopic shoulder surgery. In the current case, the origin of the embolus was unknown, but with a CT angiogram and D-dimer test, PE was diagnosed, even though a Doppler ultrasound showed no evidence of DVT in any extremity.

There is little evidence regarding the risk of and best methods to prevent VTE (either DVT or PE) associated with elective arthroscopic shoulder surgery. Different therapeutic strategies including thrombolytic therapy, surgical embolectomy, and anticoagulant medications have been recommended and used to reduce the mortality caused by PE. Using intravenous or oral anticoagulant medications is not without risk. The use of therapeutic anticoagulation increases bleeding episodes, specifically surgical site hematoma formation, therefore, is not suited to outpatient surgery or for short periods of treatment as it may take up to five days before a stable antithrombotic effect is achieved.

Muntz et al.^34^ and Hingorani et al.^35^ reported that using anticoagulation could prevent clot propagation, to facilitate the maintenance of venous collaterals and prevent PE. Thromboembolism after shoulder arthroscopy has been reported despite prophylaxis with low dose heparin.^23^ Randelli et al.^16^ surveyed 59 surgeons about 9,385 shoulder arthroscopies, concluding that twenty surgeons (33.9%) used chemoprophylaxis routinely; six DVTs were reported. Jameson et al.^12^ reported on 65,302 shoulder arthroscopies over 42 months after the institution of British national thromboprophylaxis guidelines. Results for both the DVT and PE rates were 0.01%, which was similar to that of the population at large.

PE that develops after upper extremity surgery arises mainly from the ipsilateral axillary subclavian venous system, either of the lower extremities, or on the contralateral axillary vein.^15^,^36^ In our case, the PE involved the medial segment of the right middle lobe of lung. Cortés et al.^21^ stated that ipsilateral venous injuries have been associated with venous irritation or compression by the shaver, subcutaneous edema around the shoulder by the extravasation of irrigation fluid, excessive arm traction, and inadequate positioning of the arm.

Several risk factors contribute to the development of PE following shoulder arthroscopic surgeries, including age, cancer history, hereditary thrombophilia, personal or family history of thromboembolism, tobacco abuse, diabetes mellitus, and obesity.^1^,^11^,^17^,^18^,^23^,^26^,^37^ Our patient had several risk factors including long operative time, obesity, and inflammatory bowel disease (IBD). A prolonged surgical time, caused by the difficulties in repairing the bony Bankart lesion, may have been one factor. Individuals with IBD have been shown to have an approximately 3-fold risk of DVT or PE compared to the general population.^38^ This risk correlates positively with active disease and degree of colonic involvement.^39^ There have been reports, however, of DVT and PE after shoulder arthroscopy with no discernable intrinsic risk factors in the patient.^14^

Some authors have speculated about the roles that patient positioning and traction play in increasing VTE risk in shoulder arthroscopy.^21^ There have been reports of VTE after shoulder arthroscopy in both the beach chair and lateral decubitus positions, with and without traction.^14^–^16^,^21^,^22^,^40^ Kuremsky et al.^13^ performed large retrospective series of arthroscopy in 1,908 patients over a 5-year period, and all were done in the lateral decubitus position with traction. Their result showed a VTE rate of 0.31%. This casts doubt on the role lateral positioning and traction play in VTE during arthroscopy; a definitive answer, however, did not appear to exist in the literature.

## Conclusion

Thromboembolic events after shoulder arthroscopy are rare events, but may prove life threatening. Current guidelines do not recommend the use of routine DVT chemoprophylaxis for shoulder arthroscopy patients. Surgeons should be aware of predisposing factors, various signs and symptoms with which thromboembolism may present in their patients to facilitate an early diagnosis and timely treatment when symptoms of thromboembolism arise; however, additional research for clinical validation is required.

## Informed Consent

Institutional Review Board (IRB) approval was not required for this case report; however, the patient was informed that data about the case would be submitted for publication. The patient agreed and informed consent was signed.
